# An alternative technique to safely close the chest after placing a child on central venoarterial extracorporeal membrane oxygenation

**DOI:** 10.1002/ccr3.926

**Published:** 2017-03-29

**Authors:** Dimitrios Bobos, Meletios A. Kanakis, Sofia Koulouri, Spyridon Rammos, Andreas Karabinis, Nicholas M. Giannopoulos

**Affiliations:** ^1^ Department of Pediatric and Congenital Heart SurgeryOnassis Cardiac Surgery CenterAthensGreece; ^2^ Department of Pediatric CardiologyAthens Medical CenterAthensGreece; ^3^ Department of Pediatric CardiologyOnassis Cardiac Surgery CenterAthensGreece; ^4^ Department of Internal MedicineOnassis Cardiac Surgery CenterAthensGreece

**Keywords:** Child, extracorporeal membrane oxygenation, sternum, wound closure techniques

## Abstract

Central venoarterial (VA) placement of extracorporeal membrane oxygenation (ECMO) is performed surgically, and in the majority of cases, the patient remains with an open sternum. Herein, a case of a 3‐year‐old patient who underwent insertion of a central VA ECMO for heart failure due to acute myocarditis is described. An alternative technique for ECMO placement providing sternal closure and minimizing infection risk for the safe patient transport is described.

## Case History

Question: Is it possible to place a central venoarterial (VA) extracorporeal membrane oxygenation (ECMO) on a child and subsequently closing the sternum by safely anchoring the cannulas?

Answer: We present an alternative setup for central VA ECMO placement, allowing sternal closure and safe anchoring of cannulas for safely transfer of this young patient.

A 3‐year‐old boy referred to our department for the management of cardiac failure due to the presence of acute myocarditis. The patient had a progressive clinical deterioration, and it was he who was imperative to be transferred to a specialized center of abroad for further management. The boy was placed on ECMO. Beyond patient's stabilization, the goal of this management was anchoring of cannulas and sternal closure to provide safe transport of the patient by minimizing the risk of infections. Due to lack of appropriate cannulas for access through the neck and recognizing the emergency of the case, we decided to proceed with the following alternative technique. Additionally, with the following technique, we could easily perform left ventricular vent.

**Figure 1:**
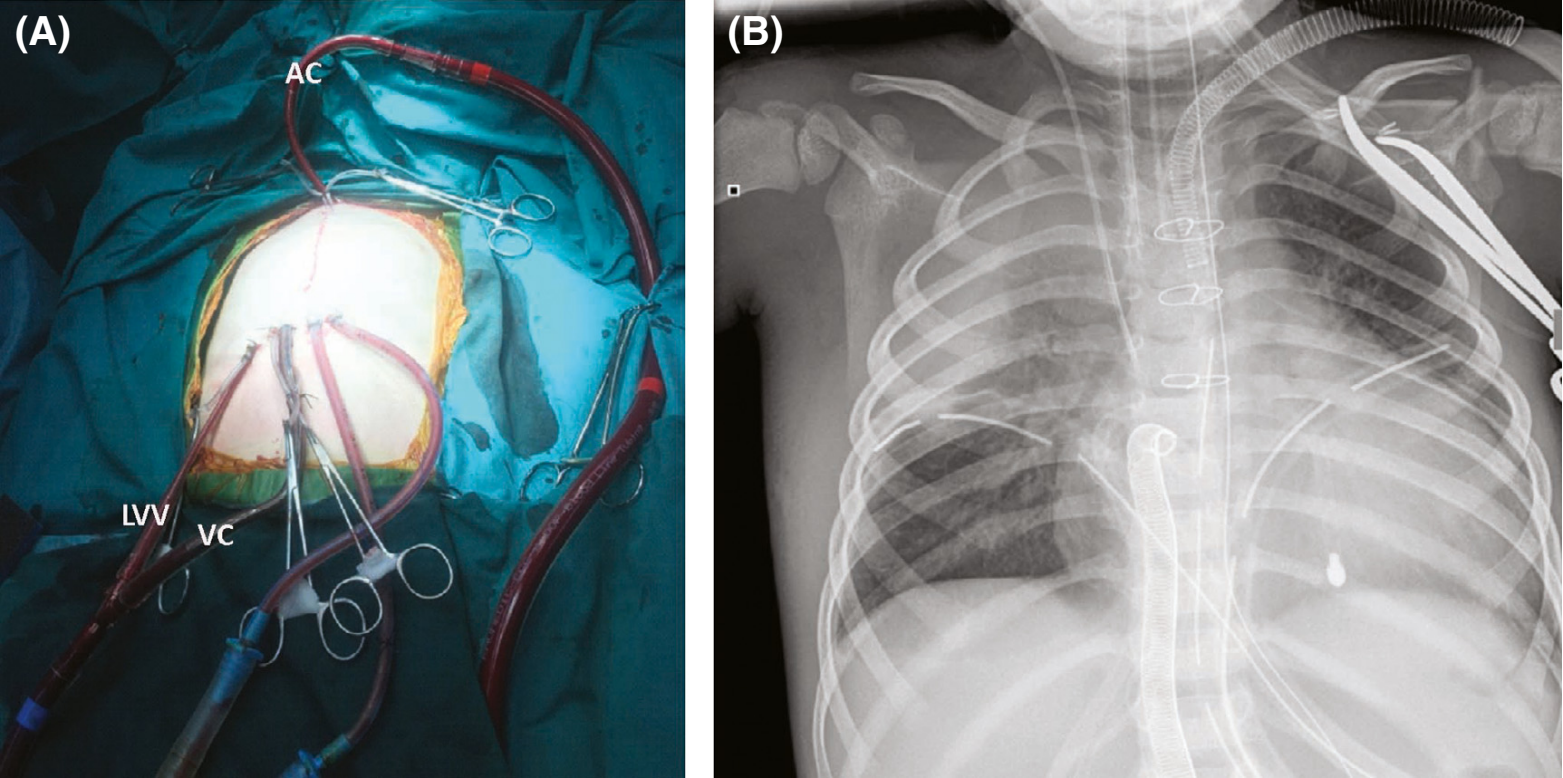
Left ventricular vent (LVV) and arterial venous cannula (VC) exit from an opening through the right subcostal margin, while arterial cannula (AC) exits through the suprasternal notch (A). Chest X‐ray showing the sites of cannulas and sternal closure with wires (B).

Both left ventricular vent and arterial cannulas exited from an opening through the right subcostal margin, while arterial cannula exited through the suprasternal notch (Fig. 1A). Chest X‐ray shows the sites of cannulas and sternal closure with wires (Fig. 1B).

The boy was safely transferred to a specialized center of abroad after 10 days of ECMO support. He finally underwent biventricular assist device support and tracheostomy and is listed for heart transplant.

## Authorship

DB: had the main idea for this alternative cannulation setup and helped to draft the manuscript. MK: participated in the main idea for this alternative cannulation setup and in the design of the manuscript and drafted the manuscript. SK: helped to draft the manuscript. SR: participated in the design of the manuscript. AK: participated in the design of the manuscript. NG: conceived of the manuscript idea and participated in its design. All authors read and approved the final manuscript.

## Conflict of Interest

None declared.

